# Supplementary figures and images for: Vesicular trafficking permits evasion of cGAS/STING surveillance during initial human papillomavirus infection
Source: PLoS Pathog. 2020 Nov 30;16(11):e1009028. doi: 10.1371/journal.ppat.1009028 (PMC7728285; doi:10.1371/journal.ppat.1009028)

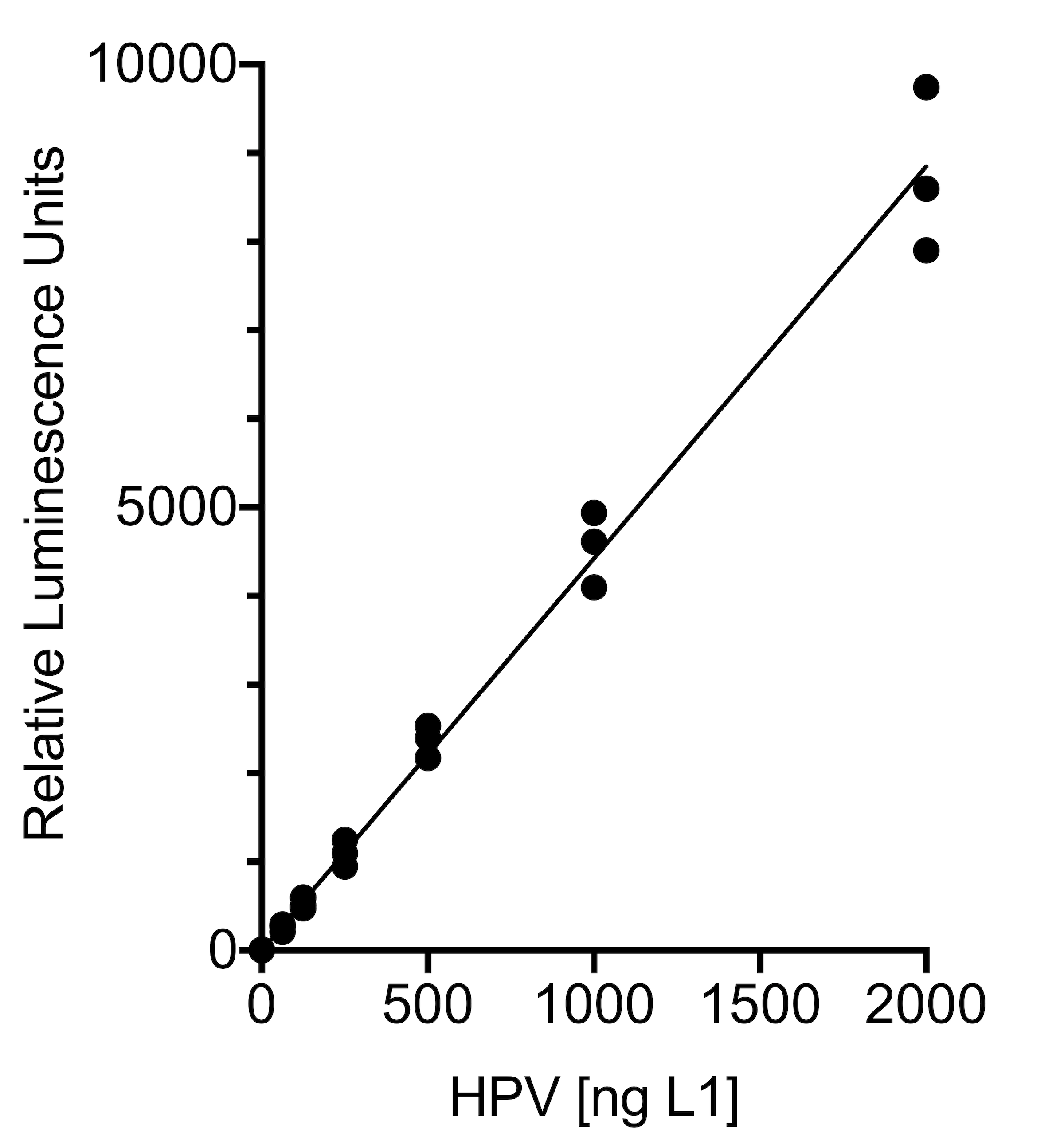

Supplement: S1 Fig — HaCaT cells were infected with increasing amounts of luciferase-expressing HPV16 pseudovirions (62.5 ng L1/well to 2000 ng L1/well). Luciferase activity was measured 24 hr post-treatment, n = 3 technical replicates, R2 = 0.987. (TIFF) [file ppat.1009028.s001.tiff]

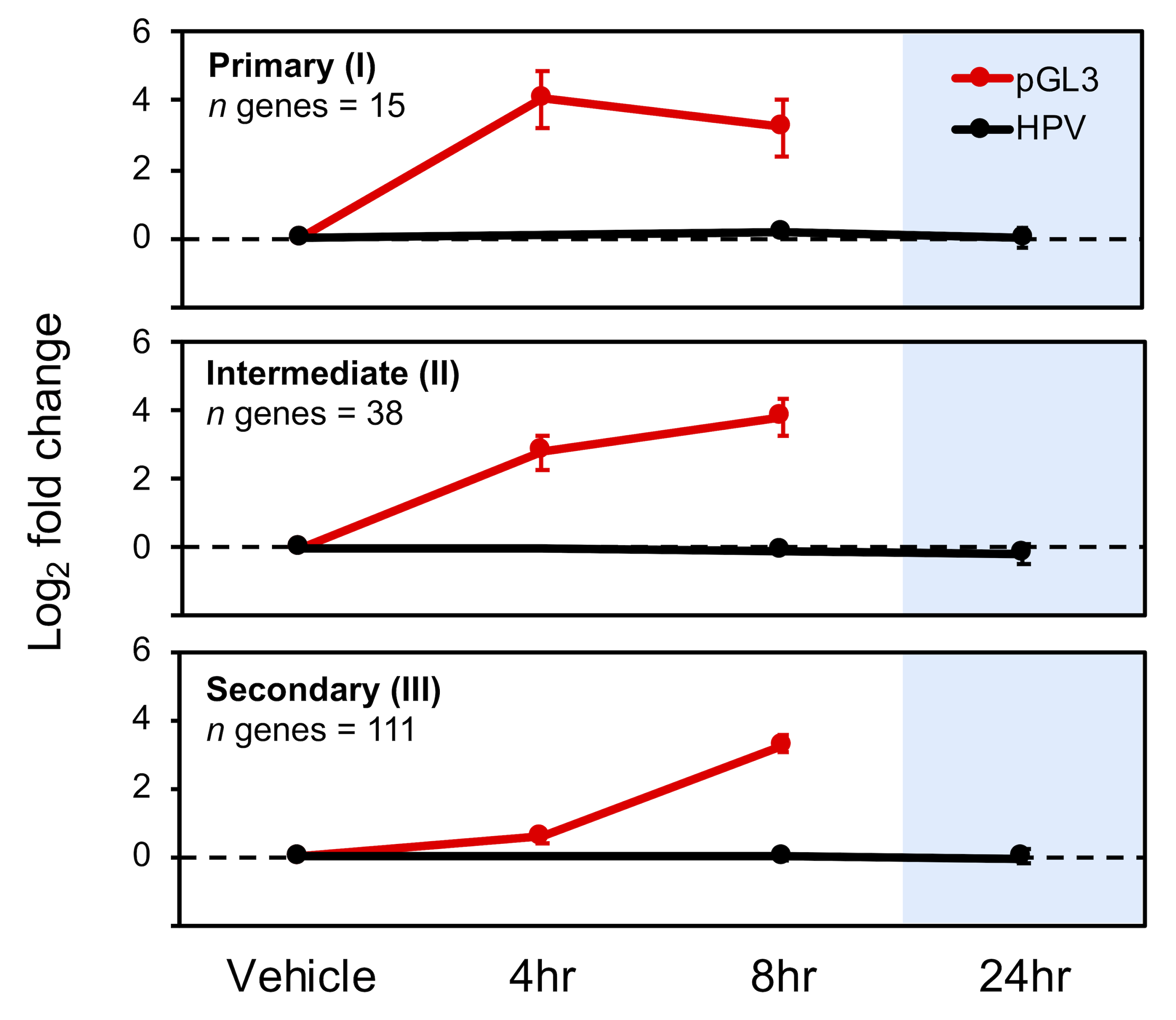

Supplement: S2 Fig — Aggregate gene responses following pGL3 DNA introduced via liposome transfection (red, n = 2 biological replicates) or HPV virion infection (black, n = 3 for Vehicle, 4hr, and 8hr). Preliminary data for 24hr post-infection (blue background, n = 1) indicate cellular responses to DNA remain inactive throughout the initial infection. Metagene log2 fold change values were computed by aggregating RNA-seq data of all genes within each distinct cellular response cluster (see Fig 3C and 3D). Error bars represent 95% confidence intervals within each gene cluster. (TIFF) [file ppat.1009028.s002.tiff]
