# Supplementary material for: Vesicular trafficking permits evasion of cGAS/STING surveillance during initial human papillomavirus infection
Source: PLoS Pathog. 2020 Nov 30;16(11):e1009028. doi: 10.1371/journal.ppat.1009028 (PMC7728285; doi:10.1371/journal.ppat.1009028)
Supplement: S2 Data — (RTF) [file ppat.1009028.s004.rtf]

Sample,SeqBatch,SampleBatch,Delivery,Cargo,Treatment,Time
Y_H2O_4,Seq1,Y,Transfection,Vehicle,H2O,4hr
Y_pGL3_4,Seq1,Y,Transfection,dsDNA,pGL3,4hr
Y_pGL3_8,Seq1,Y,Transfection,dsDNA,pGL3,8hr
Z_H2O_4,Seq1,Z,Transfection,Vehicle,H2O,4hr
Z_pGL3_4,Seq1,Z,Transfection,dsDNA,pGL3,4hr
Z_pGL3_8,Seq1,Z,Transfection,dsDNA,pGL3,8hr
A_VSB_8,Seq2,A,Infection,Vehicle,VSB,8hr
A_HPV_8,Seq2,A,Infection,dsDNA,HPV,8hr
B_VSB_8,Seq2,B,Infection,Vehicle,VSB,8hr
B_HPV_8,Seq2,B,Infection,dsDNA,HPV,8hr
C_VSB_8,Seq2,C,Infection,Vehicle,VSB,8hr
C_HPV_8,Seq2,C,Infection,dsDNA,HPV,8hr
C_HPV_24,Seq2,C,Infection,dsDNA,HPV,24hr
